# Supplementary material for: Nucleotide sugar biosynthesis occurs in the glycosomes of procyclic and bloodstream form Trypanosoma brucei
Source: PLoS Negl Trop Dis. 2021 Feb 16;15(2):e0009132. doi: 10.1371/journal.pntd.0009132 (PMC7909634; doi:10.1371/journal.pntd.0009132)
Supplement: S1 Fig — Analysis by SDS-PAGE and Coomassie blue staining of TbMPGT expressed in E.coli, purified by nickel chromatography, digested with thrombin to remove the N-terminal 6-His tag and then purified by FPLC gel-filtration. Molecular weight standards are shown on the left. (DOCX) [file pntd.0009132.s001.docx]

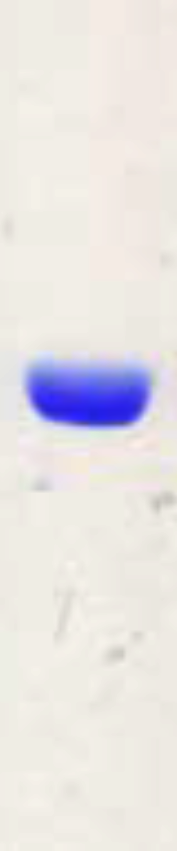


**S1 Fig.**. **Recombinant TbMPGT.**

Analysis by SDS-PAGE and Coomassie blue staining of TbMPGT expressed in *E.coli*, purified by nickel chromatography, digested with thrombin to remove the N-terminal 6-His tag and then purified by FPLC gel-filtration. Molecular weight standards are shown on the left.
