# Supplementary material for: Nucleotide sugar biosynthesis occurs in the glycosomes of procyclic and bloodstream form Trypanosoma brucei
Source: PLoS Negl Trop Dis. 2021 Feb 16;15(2):e0009132. doi: 10.1371/journal.pntd.0009132 (PMC7909634; doi:10.1371/journal.pntd.0009132)
Supplement: S2 Fig — The mouse polyclonal antibodies raised against TbMPGT, TbGNA, TbUAP, TbGALE and TbGMER (Tables 1 and 2) were used in Western blotting with an anti-mouse-HRP secondary antibody. The lanes contained either 5 x 10^6^ cell equivalents of T. brucei bsf total cell lysates (lanes 1, 2, 3, 5, and 7) or, when no signal was recorded against whole lysate due to low abundance, the lanes contained an immunoprecipitate from 2 x 10^8^ cell equivalents of total bsf lysate using rabbit anti-TbGALE [44] (lane 4) or mouse anti-TbGMER (lane 6). In each case, a single band* with an apparent molecular weight consistent with the target antigen was recorded, demonstrating the mono-specificity of the antibodies. *Note: the two additional bands marked by asterixis in (lane 6) are due to mouse IgG heavy and light chains from the immunoprecipitation. A Ponceau red stain of a Western blot lane containing 5 x 10^6^ cell equivalents of T. brucei bsf total cell lysate is shown in (lane 8). The positions of MW standards are shown on the right. (DOCX) [file pntd.0009132.s002.docx]

**S2 Fig. Mono-specificity of antibodies to nucleotide sugar biosynthetic enzymes used in this study.**

The mouse polyclonal antibodies raised against TbMPGT, TbGNA, TbUAP, TbGALE and TbGMER (Tables 1 and 2) were used in Western blotting with an anti-mouse-HRP secondary antibody. The lanes contained either 5 x 10^6^ cell equivalents of *T. brucei* bsf total cell lysates (lanes 1, 2, 3, 5, and 7) or, when no signal was recorded against whole lysate due to low abundance, the lanes contained an immunoprecipitate from 2 x 10^8^ cell equivalents of total bsf lysate using rabbit anti-TbGALE [44] (lane 4) or mouse anti-TbGMER (lane 6). In each case, a single band* with an apparent molecular weight consistent with the target antigen was recorded, demonstrating the mono-specificity of the antibodies. *Note: the two additional bands marked by asterixis in (lane 6) are due to mouse IgG heavy and light chains from the immunoprecipitation. A Ponceau red stain of a Western blot lane containing 5 x 10^6^ cell equivalents of *T. brucei* bsf total cell lysate is shown in (lane 8). The positions of MW standards are shown on the right.
