# Supplementary material for: Nucleotide sugar biosynthesis occurs in the glycosomes of procyclic and bloodstream form Trypanosoma brucei
Source: PLoS Negl Trop Dis. 2021 Feb 16;15(2):e0009132. doi: 10.1371/journal.pntd.0009132 (PMC7909634; doi:10.1371/journal.pntd.0009132)
Supplement: Attachment — Submitted filename: PLoS NTD paper replies to reviewers_261020.docx [file pntd.0009132.s003.docx]

**Replies to Reviewer’s Comments**

We are very grateful to the reviewers for their careful reading of the manuscript and their helpful and constructive comments which we address, *marked in italics with >>>,* as follows:

**Reviewer 1**

**Major points:**

1. In lines 710-712 the authors state that a portion of the figure has been reproduced from another manuscript.  I've not seen this done before and am unsure how to evaluate this data. Perhaps the authors could state why they are using previously published work instead of data from their own experiments.  Also, it would be helpful if the authors identified these data visually in the figure as I wasn't able to discern exactly which parts were previously published. Is this portion of the figure necessary to show that digitonin is solubilizing cell membrane but that glycosome components are still protected? If so, the enolase and aldolase panels are essential for those controls.  Can the authors clarify this issue?

*>>>We apologise for the confusion this seems to have caused, although what we did is in-line with PLoS NTD guidelines, with appropriate copywrite permissions etc. However, we appreciate it is unusual and to clarify:*

- *These data are our own data (performed by Dr M. Lucia S. Guther).*
- *The experiments (for TbUAP, TbGalE, TbUAP, TbPMI, TbPMM, TbMPGT, TbGMER, and TbPEX13) were performed at the same time as the data reported in Bandini, Guther….and Ferguson ref. 42 for TbPAGM, TbAldolase and TbEnolase.*
- *We include the TbPAGM data here again for completeness and we include the shared controls (TbAldolase and TbEnolase) that go with this entire batch of data.*
- *To make this clear we now provide boxes around the data that appeared previously in our previous work (ref 42) and have added some additional text to the Fig. 3 legend: “Note: The data in Panel A were collected at the same time as data reported in Figure 8C of reference [42] (boxed). These are included for completeness with permission from John Wiley and Sons (licence 4846630270803).”*

2. The explanation regarding the previous finding of TbMPGT in the cytoplasm is less compelling as the authors suggest the discrepancy is due to using antibodies raised against Leishmania MPGT (Denten et al. PMID:19919534).  While the IFA signal in the Denten et al paper may have been a result of non-specific interactions (there is no control with MPGT-deficient parasites), the fractionations indicate that most of the signal was found in the cytosolic fraction with a small amount found in microsomes.  While there does appear to be a difference in size between the cytosolic and microsomal species the westerns yield a single, well-defined band. Is there another explanation? Is it possible that this protein could exhibit dual localization under different growth conditions or in different strains where perhaps import of the protein is inefficient?

*>>>The same strain of trypanosomes (427) was used. The intactness of the glycosomes was not demonstrated by Denton et al nor was a counter-stain (Western or IFA) performed with an authentic glycosomal and/or cytoplasmic marker(s). Nevertheless, we add a comment that: “….it is possible that different growth conditions or other factors might account for this discrepancy”*

3. In lines 164-166 the authors state that aldolase and GAPDH are not quantitatively released until addition of Triton because of the "aggregated nature of these very abundant proteins in the dense crystalloid glycosome core" While many have speculated that the dense nature of the glycosomes is responsible for some of the unique behaviors of glycosome proteins, this hasn't been experimentally demonstrated.  For this reason, it may be desirable to qualify the statement to "it has been proposed that ....".  I agree that this "non-quantitive" release is common but I don't know that the cause of this is understood.

*>>>We thank the reviewer and agree and have modified the text as recommended. It now reads: “…but because of the possibly aggregated nature of these very abundant proteins in what has been proposed to be a dense crystalloid core inside of glycosomes [56].*

**Minor Points**

1. Qualify the statement in lines 163-166 to highlight that people have hypothesize that the non-quantitative release often observed with glycosome proteins is due to the aggregated nature of these proteins. *>>>see immediately above for Major Point 3.*
2. --Ln 286 should read "His-tag"?*>>>Yes, corrected*
3. --Lines 308,310: OD280 formatting*>>>defined and corrected*.
4. --Line 314-TBD buffer composition is not given*>>>now added*
5. --Line 682 T. brucei should be italicized*>>>corrected*
6. --Lines 710-712 and Figure 3: clarify which regions of the figure were reproduced from previous work*>>>see new Figure 3 and comments above for Major Point 1.*
7. --Figure 3--red box around text*>>>Removed*

**Reviewer 2:**

**Major points:**

1) The images in Figure 2 appear to be maximum intensity projections. Planar slices should be shown. Moreover, quantification should be performed and the quantification data presented to show the amount of colocalization between a particular enzyme and the glycosomal marker GAPDH*.*

*>>>The data shown are planar slices and quantitative data have now been added in Table 3. These quantitative data are also described now in the Results and in the Discussion.*

2) Subcellular fractionation must be done to show that a particular enzyme cofractionates with a known glycosomal marker and not with markers of other organelles such as the mitochondrion or ER*.*

*>>>We respectfully disagree. With the properly controlled (i.e., with authentic glycosome and cytosol markers) IFM and* *digitonin latency experiments we believe we provide more data than most glycosomal location claims. Subcellular fractionation is rather seldomly practised for subcellular localization in trypanosomes these days (rightly or wrongly). Where previous subcellular fractionation data are available, we have noted these in Table 1.*

3) Fig. 3A. As stated on lines 710-712, images in Figure 3A are taken from a previous publication. This is not acceptable. The authors should repeat the experiments.

*>>>Again, we apologise for the confusion but refer to our response to Reviewer 1, Major point 1.*

**Minor points:**

1) Add scale bars to Figure 2*.>>>These have been added*

2) Figure S1 can be removed*.>>>With respect, we would prefer to keep the supplementary figure. Since there are discrepancies in TbMPGT localisation data, we think that showing the purity of the immunogen used in our study has some value.*

3) Line 76. “... data suggest...” NOT “...data suggests...”.*>>>Corrected, thank you.*

4) Lines 158 and 159. There is some controversy that TbPEX13.1 is transmembrane. Best to rephrase simply as ‘membrane’.*>>>Modified, as requested.*

5) Line 175. The word ‘notion’ is inappropriate. Replace with ‘evidence’ and remove the ‘The’.*>>>Modified, as requested.*

6) Line 215. ‘pulse-chase’ NOT ‘label-chase’.*>>>We apologise for any confusion. This is a stable-isotope label (to steady state) - chase experiment and not a pulse (which by inference is not to steady state) – chase experiment. We have re-written the text as follows to provide a much better description: Evidence for the latter can be found in label-chase proteomic studies [54]. In this work, cells expressing GFP-tagged Pex13.1 were labelled to steady-state over 16 h with heavy isotope lysine and arginine and then placed into medium containing light lysine and arginine to perform a 5 h isotopic chase. Immediately after steady-state (heavy) labelling and after (light) chase, magnetic beads bearing anti-GFP antibodies were used to capture Pex13.1 containing organelles which were taken for stable isotope in cell culture (SILAC) quantitative proteomics. At the steady-state label time point, Pex13.1 was found associated with several ER resident proteins as well as glycosome components. However, after the light isotopic chase period, the apparent association of Pex13.1 with ER proteins was greatly diminished, suggesting that Pex13.1 and ER resident proteins part company during the biogenesis of glycosomes.”*

7) Line 217. ‘chase away’ is not scientific. Rephrase*.>>>Agreed, see response to point 6, above.*

8) Line 219. “The same group has...” NOT “the same group have...”.*>>>Corrected*

9) Line 229. “...which converts...” NOT “...that converts...”.*>>>Corrected*

10) Line 681. ‘mutase’ NOT ‘mutate’. *>>>Corrected*

11) Line 698. “...forms of T. brucei.” NOT “...form T. brucei.”*>>>Corrected.*

**Reviewer3:**

**Major Points:** None

**Minor Points:**

1) Since the bulk of the evidence in this manuscript relies on the immunofluorescence localization studies, the inclusion of more representative images is a must. The evaluation of subcellular distribution and colocalization with GAPDH based on just one image per NS biosynthetic enzyme is inadequate. Moreover, given the considerable amount of non-overlap between GAPDH and the nucleotide sugar enzymes, this seems particularly important.

*>>>We appreciate this comment and although we continue to use a single cell images in Figure 2 to have sufficient magnification for the reader to assess colocalization, we also now include wider-field images showing several cells as requested in figshare* (10.6084/m9.figshare.13124909)*. We trust this is an acceptable solution. Further, we have addressed the degree of overlap in the quantitation data in Table 3 (see also response to Reviewer 2 Major Point 1) and describe this in more detail in the Results and in the Discussion.*

2) Along those lines, it appears from the images that some of the NS biosynthetic enzymes show a minor distribution adjacent to or even along the flagellum, and this appears distinct from GAPDH. Can the authors comment on whether this is the case or perhaps an artifact of the images shown here?

*>>>We think this is an artefact of the wide range of NS enzyme : TbGAPDH signal ratios (evident from Table 3) and this is now discussed.*

3) I agree that the data certainly indicate glycosome colocalization for the NS biosynthetic enzymes, however, the distinct distribution from GAPDH is intriguing. While the authors indicate this is likely due to glycosome heterogeneity, I am curious whether the authors have looked at other markers? Either glycosomal or perhaps other subcellular compartments?

*>>>Not specifically, but the punctate labelling patterns are very distinctive from, for example, typical nuclear, ER, Golgi, lysosome/endosome, mitochondrial patterns.*

4) For the digitonin latency studies with BSF and PCF parasites described in Figure 3 A and B, the distribution of TbPAGM seems more distinctly glycosomal in PCF parasites. Also, I may be interpreting the figure legend incorrectly, but it suggests that the bottom two control panels for the BSF digitonin experiments, aldolase and enolase, were included from previous work? I don't consider data generated in a previous experiment to be an adequate control for use in this experiment.

*>>>Please see replies to Reviewer 1, Major Point 1.*

5) Typographical error ln 300, TbUGP should read TbUAP*.>>>Corrected, thank you.*
