# Supplementary material for: Nucleotide sugar biosynthesis occurs in the glycosomes of procyclic and bloodstream form Trypanosoma brucei
Source: PLoS Negl Trop Dis. 2021 Feb 16;15(2):e0009132. doi: 10.1371/journal.pntd.0009132 (PMC7909634; doi:10.1371/journal.pntd.0009132)
Supplement: Attachment — Submitted filename: PLoS NTD paper replies to reviewers_241220.docx [file pntd.0009132.s004.docx]

**Replies to Reviewer’s Comments**

We are very grateful to the reviewers for their careful reading of the revised manuscript and their helpful and constructive comments which we address, *marked in italics with >>>,* as follows:

**Comments on revision 1:**

We thank you for the revisions addressing many of the reviewers issues*.* However, at this time it will not be possible to accept the manuscript in its current state since a critical point raised by by two reviewers with extensive cell biology and organelle biogenesis were not addressed. This is an important issue since the major thrust of the manuscript is to assign the localization of NS biosynthesis enzymes. Given the fact that the localization of some NS biosynthesis enzymes have been previously reported to have an incorrect localization based on IFM studies ist is critical to validate the findings that you report in this manuscript using subcellular localization as recommended by Reviewer #2 and that additional marker enzymes for ER and mitochondria should be included*.*

*>>>In the sub-cellular fractionation Western blots (Fig 6A)* ***we now show the distribution of authentic mitochondrial and ER markers*** *(as well as authentic glycosome and cytoplasmic markers).*

The need for these additional experiments is further emphasized by the issue raised by Reviewer # 3, "Along those lines, it appears from the images that some of the NS biosynthetic enzymes
show a minor distribution adjacent to or even along the flagellum, and this appears distinct from GAPDH. Can the authors comment on whether this is the case or perhaps an artifact of the images shown here?" The rebuttal to this comment 
">>>We think this is an artefact of the wide range of NS enzyme : TbGAPDH signal ratios (evident from Table 3) and this is now discussed." is a little concerning as does not sufficiently address the reviewer's concerns. It is acknowledge that IFM and digitonin latency  is frequently used (right or wrong), a more robust approach would be to include a subcellular fractionation experiment as indicate by the Reviewers. A careful examination of the Reviewer comment indicate that a subcellular fractionation experiment was a MUST*.>>>see below.*

*>>>In this latest revised version, we directly address the reviewer’s requests for subcellular fractionation data. We provide this in Fig 6A for TbPMI, TbMPGT and TbUGP along with markers for the glycosome, the cytoplasm* ***and the mitochondrion and the ER*** *(as specifically requested). Because several of the antibodies in this paper were raised some time ago and have since been exhausted, we are unable to perform sub-cellular localisation for every one of NS biosynthetic enzymes. However, as shown in Table 1, there are 3 other NS biosynthetic enzymes (TbHK, TbPGI and TbGALE) that have also been localized the glycosomes in published studies, bringing the total to 6.*

*We sincerely hope that the sub-cellular localisation data on three representative NS biosynthetic enzymes (TbPMI, TbMPGT and TbUAP) – which places them squarely in the small granular (glycosomal) fraction - together with the 3 previously published sub-cellular localisation examples, and our extensive immunolocalization and digitonin latency data, will be sufficient to convince the reviewers that NS biosynthesis occurs in the glycosomes of T. brucei.*

*Please also note that we now include some density gradient fractionation and label-free quantitative proteomics data that we feel adds significantly to the discussion on glycosome heterogeneity (Fig 6B and C).*

*We are grateful for the review process.*
